# A Rare Case of Cardiac Myxoma With Moyamoya Phenomenon: A Disease or Syndrome?

**DOI:** 10.7759/cureus.59381

**Published:** 2024-04-30

**Authors:** Rayees A Konduru, Ankita Prasad, Pramil Cheriyath, Arthur Okere

**Affiliations:** 1 Anesthesiology, NewYork-Presbyterian Brooklyn Methodist Hospital, New York, USA; 2 Pediatrics, NewYork-Presbyterian Brooklyn Methodist Hospital, New York, USA; 3 Internal Medicine, Saint Clare’s Denville Hospital, Denville, USA

**Keywords:** moyamoya disease, moyamoya phenomenon, embolization, cardiac myxoma, fragile network of blood vessels, proangiogenic factors, stroke, intracerebral hemorrhage, collaterals, moyamoya syndrome

## Abstract

Moyamoya disease (MMD) is a rare, idiopathic, progressive, obstructive, vasculopathy affecting primarily the terminal portions of the intracerebral internal carotid arteries, typically at the base of the brain. It is more commonly seen in people of East Asian descent. The moyamoya phenomenon refers to the characteristic appearance of the tangle of fine blood vessels, also described as a puff of smoke. Moyamoya syndrome (MMS) refers to the constriction-induced chronic brain ischemia that is believed to cause overexpression of proangiogenic factors, creating a fragile network of collateral capillaries. MMS refers to the moyamoya phenomenon in the presence of other congenital or acquired disorders. Intracerebral hemorrhage is the leading cause of death for MMS patients. Overall, the prognosis is variable. Cardiac myxoma can cause embolization of tumor cells, plaques, and thrombus, and recurrent thromboembolism can lead to chronic brain ischemia, which can lead to the development of collaterals. There have been cases reported where the moyamoya phenomenon resolved following myxoma resection. Here, we present the case of a female who had intraventricular bleeding and was diagnosed with MMD. Eighteen months later, she presented with shortness of breath and was diagnosed with cardiac myxoma with multiple valvular regurgitations. The myxoma was surgically removed.

## Introduction

Moyamoya disease (MMD) is a progressive obstructive vasculopathy of idiopathic origin affecting primarily the terminal, typically at the base of the brain. The term “moyamoya” is of Japanese origin, where this disease was first described, and means a puff or wisp of smoke. Moyamoya syndrome (MMS) describes the same vascular changes as MMD but is associated with inherited or acquired conditions that can cause chronic ischemia or vasculopathy in the brain. The Moyamoya phenomenon refers to the characteristic appearance of the tangle of fine blood vessels formed in response to chronic ischemia. Constriction-induced chronic brain ischemia creates a fragile network of collateral capillaries [1]. It mainly presents as stroke or transient ischemic attacks, and intracerebral hemorrhage is the leading cause of death for MMD patients [2]. MMD is classified into four subtypes: ischemic, hemorrhagic, epileptic, and other. Ischemic stroke is more prevalent than hemorrhagic stroke in children [3]. MMD may be linked to chromosomes 17 and 3.

Overall, the prognosis is variable [4]. Over five years, two-thirds of patients with MMD experience clinical progression and bad prognosis [3]. The two peak age groups affected by this condition are 5-9 years and 45-49 years [3]. It occurs mostly in people of East Asian origin, especially in Japan and Korea. MMD is more common in females than males, with a female-to-male ratio of 2.2 [3].

## Case presentation

**Figure 1:**
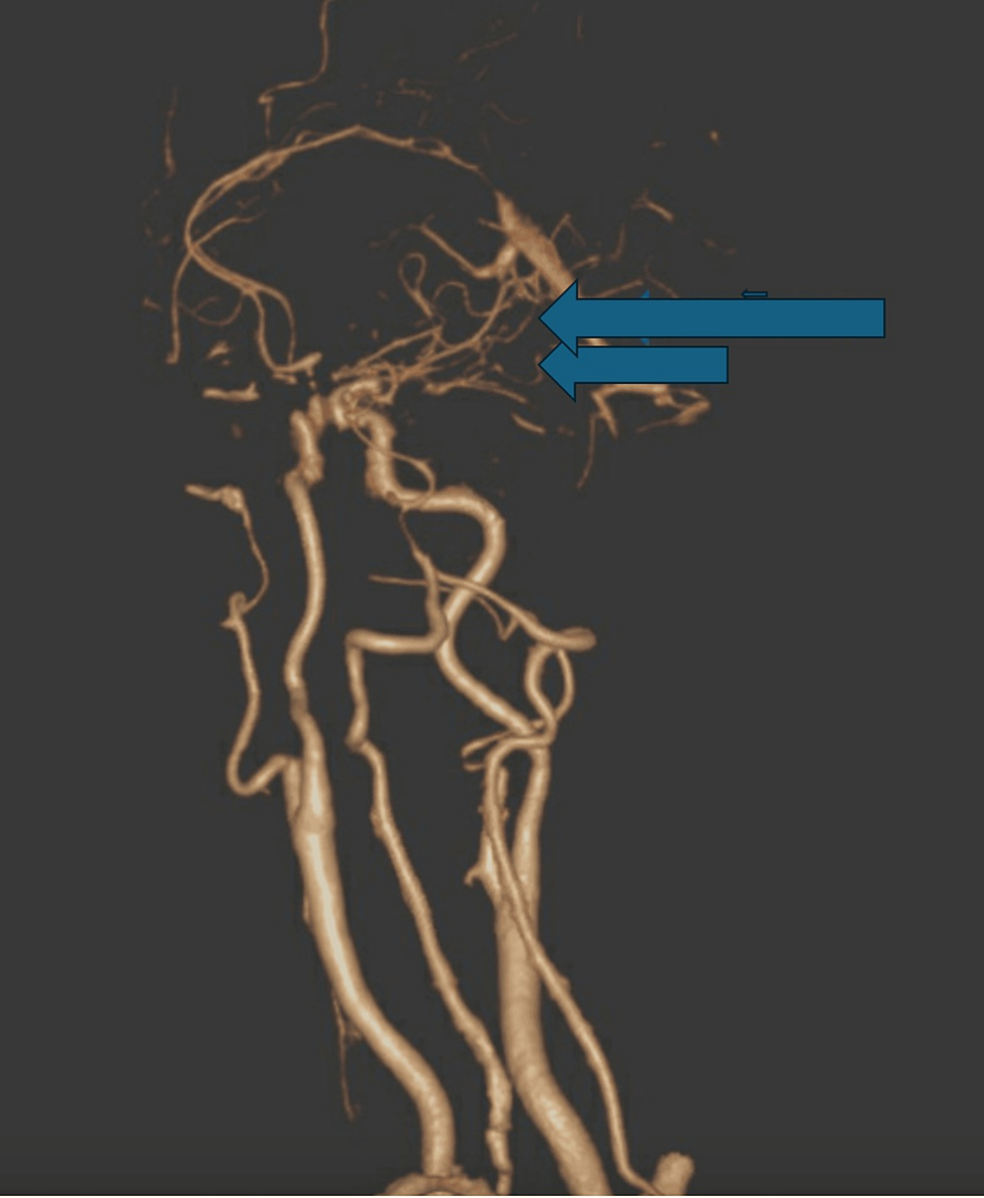
CT angiography showing wispy collateralization characteristic of moyamoya disease (blue arrows).

A 65-year-old woman from the Philippines presented to the hospital with new-onset shortness of breath and off-and-on palpitations for over two months. Her shortness of breath had gradually worsened, and she was getting breathless with her activities of daily living. She was being evaluated for these symptoms in the outpatient setting. There was no associated fever, cough, chest pain, loss of consciousness, headache, vomiting, or leg or abdominal swelling. Her medical history was significant for MMD, which was diagnosed in the Philippines one year ago, and a prior angiography showed bilateral collateralization (Figure 1).

One year prior, she was hospitalized for an intraventricular hemorrhage following severe headache and dizziness. At that time, she recovered without sequelae. The transthoracic echocardiography done at that time as a part of her workup showed no intracardiac pathology. She also had a history of atrial fibrillation, paroxysmal supraventricular tachycardia, and rheumatic heart disease with severe mitral stenosis, and a mitral valve commissurotomy was done a year ago. She also had hypertension and hyperlipidemia and was on aspirin, atorvastatin, and metoprolol. She also had intermittent leg claudication and was on an exercise program. She was a nonsmoker and nonalcoholic, and her family history was noncontributive.

**Figure 2:**
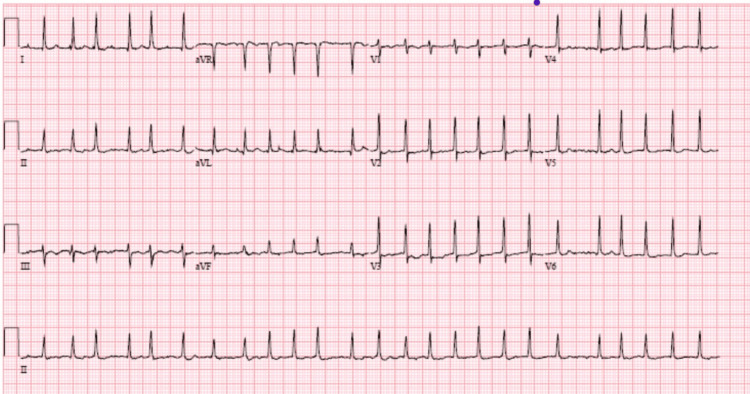
EKG showing atrial fibrillation with a rapid ventricular rate (154 beats/minute).

At the presentation, she was conscious and alert. Her pulse was irregular, and her heart rate was 154 beats per minute. Her blood pressure was 132/84 mmHg, her respiration rate was 20 breaths per minute, and her oxygen saturation was 95% at room air. Her EKG showed atrial fibrillation (Figure 2).

**Figure 3:**
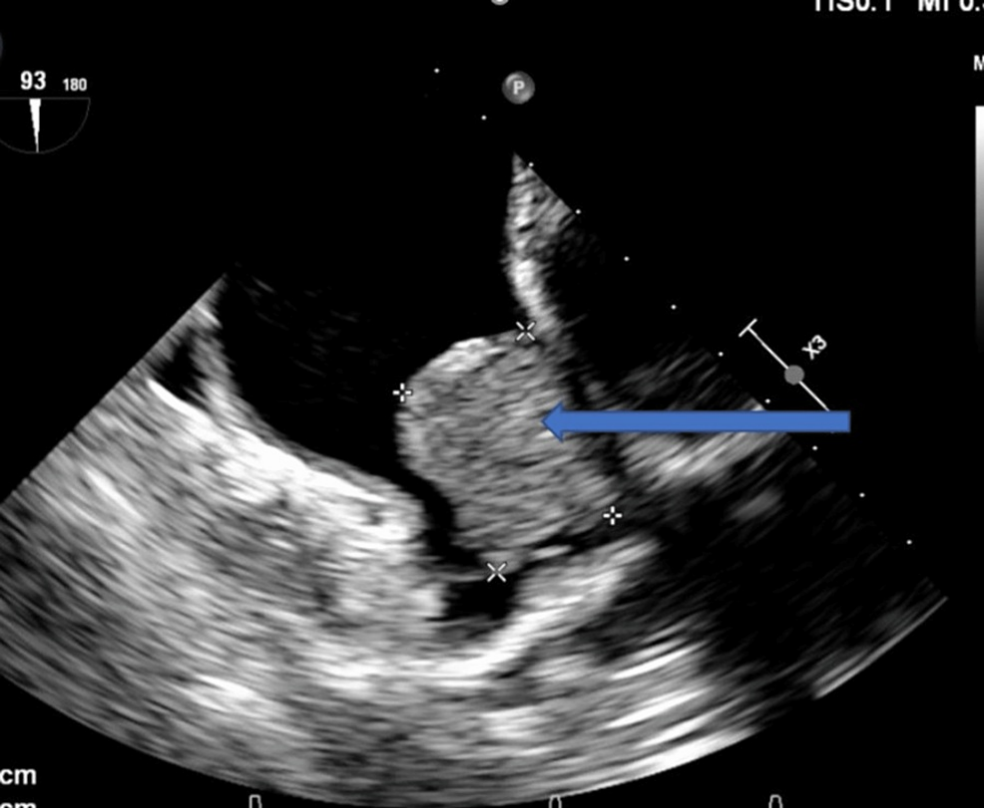
Transesophageal echocardiography showing myxoma attached to the left atrium (blue arrow).

Blood tests included a complete blood count and metabolic panels. Her troponin level was 0.05 ng/mL (0.04 ng/mL), and her brain natriuretic peptide level was 350 pg/mL (<100 pg/mL). Her transesophageal echocardiogram showed a large, fixed mass measuring 3.6 cm in the left atrium, suspected of atrial myxoma (Figure 3).

The ejection fraction was 50-55%. It also showed mild mitral regurgitation, moderate-to-severe tricuspid regurgitation, moderate aortic valve regurgitation, no pericardial effusion, and no atherosclerotic plaque in the aorta. She had a left heart catheterization, which revealed no coronary artery disease. She was taken for cardiac surgery and left atrial myxoma removal, and mitral valve replacement was done successfully.

## Discussion

MMS and MMD have the same vascular pathology and clinical and imaging features. However, MMS is associated with other congenital or acquired conditions instead of being idiopathic [3]. MMD is characterized by the bilateral collateralization of the vessels. Unilateral collateralization is usually associated with MMS [3]. MMS can be associated with congenital and genetic conditions such as sickle cell disease, Down syndrome, aplastic anemia, Fanconi anemia, sickle cell anemia, lupus anticoagulant, Apert syndrome, Marfan syndrome, tuberous sclerosis, and Turner syndrome [5]. The most common acquired conditions seen with MMS are thyroid issues such as Graves’ disease or thyroiditis, atherosclerosis, aortic coarctation, fibromuscular dysplasia, cranial trauma, radiation injury, parasellar tumors, and infectious diseases such as leptospirosis and tuberculosis [5]. MMS is associated with mutations in the *BRCC3*, *MTCP1*, and *GUCY1A3* genes [6]. Some studies have mentioned a probable underlying immunological phenomenon [3]. Understanding the pathogenesis of MMS can provide insights into the pathogenesis of the disease. It will also help us figure out how to treat complicated illnesses associated with MMS.

A Korean study showed the risk of MDD was 132-fold higher in individuals who have first-degree relatives with MDD and the familial risk increased with the degree of genetic relatedness [7]. Mineharu et al. proposed that familial MMD is autosomal dominant with variable penetrance based on age and genomic imprinting variables and progresses regardless of symptom severity, ongoing treatment, age, gender, disease type, or disease location [4].

The pathology behind this vascular phenomenon is the thickening of the intima of the blood vessels. The disease is associated with a fibrocellular thickening of the intima with an undulating internal elastic lamina and a media thinning [3]. Bilateral stenosis or occlusion of the terminal parts of the internal carotid arteries close to the base of the brain with the characteristic appearance of the tangle of fine blood vessels forms in response to chronic ischemia [3]. Chronic brain ischemia caused by constriction leads to the overexpression of proangiogenic factors. This creates a fragile network of collateral capillaries. Previously thought to be new, the collateral vessels are now shown to be enlarged preexisting microperforator arteries such as the lenticulostriate and thalamoperforating arteries [8]. Serum studies on people with active disease show higher levels of angiogenic growth factors such as vascular endothelial growth factor, matrix metalloproteinase hepatocyte growth factor, and interleukin-1β [9]. The hypoperfused brain parenchyma recruits blood vessels from the leptomeninges as the disease worsens [8]. These blood vessels eventually connect to the external carotid artery and form anastomotic branches [8]. These newly formed collaterals are fragile and prone to aneurysmal formation and rupture because of the fragmentation of the elastic layer [8].

About 50% of all cardiac tumors are atrial myxomas originating from multipotent mesenchymal cells of the endocardium. They constitute between 50% and 83% of all primary cardiac tumors [10]. They are frequently detected in the left atrium and are sources of thrombotic and tumor emboli. Additionally, brain aneurysms can be seen in myxomas. In the literature, myxoma is rarely cited in association with moyamoya. A study of cardiac myxoma by Long and Gao [11] mentioned a case of cardiac myxoma, which subsequently caused the development of MMS. The removal of cardiac myxoma was associated with the resolution of moyamoya vessels.

Similarly, Das et al. [12] described a young adult with recurrent strokes and angiographic evidence of unilateral MMS. Etiological analysis indicated the presence of a myxoma in the left atrium. The myxoma was surgically removed, and moyamoya’s angiographic abnormalities nearly resolved afterward. moyamoya angiographic changes in myxoma may be linked to repeated embolism, post-embolic arterial wall degradation, and subsequent inflammatory response mediated by interleukin-6. This can activate a signal transduction pathway dependent, impacting the onset and rapid progression of MMS in susceptible individuals [13]. Myxoma excision surgically restored moyamoya angiographic changes, most likely due to decreasing pro-inflammatory mediators [13]. The moyamoya vascular phenomenon should also be considered a rare complication of cardiac myxoma. Our patient was diagnosed with cardiac myxoma at least 18 months after the diagnosis of MMD. We had no earlier documentation of cardiac myxoma when she was diagnosed with MMD. Hence, is it possible for an asymptomatic cardiac myxoma to throw emboli and cause the moyamoya phenomenon or was it an unrelated event? It is possible that she was actually a case of MMS and not MMD as previously thought.

## Conclusions

MMS and MMD have the same vascular pathology and clinical and imaging features. However, MMS is associated with other congenital or acquired conditions instead of being idiopathic. MMS can be due to chronic brain ischemia or an immunologic phenomenon. Cardiac myxoma is characterized by the embolization of tumor cells in the brain and can lead to the formation of moyamoya vessels on the brain due to chronic ischemia or an immunologic phenomenon. In these cases, moyamoya vessels show some resolution with the surgical removal of cardiac myxoma.
